# Sweet Cherry Diversity and Relationships in Modern and Local Varieties Based on SNP Markers

**DOI:** 10.3390/plants12010136

**Published:** 2022-12-27

**Authors:** Marino Palasciano, Diana L. Zuluaga, Domenico Cerbino, Emanuela Blanco, Gaetano Aufiero, Nunzio D’Agostino, Gabriella Sonnante

**Affiliations:** 1 Department of Soil, Plant and Food Sciences, University of Bari “Aldo Moro”, Via G. Amendola 165/A, 70126 Bari, Italy; 2 Institute of Biosciences and Bioresources, National Research Council, Via Amendola 165/A, 70126 Bari, Italy; 3 Agenzia Lucana di Sviluppo e di Innovazione in Agricoltura (ALSIA) Pollino, C.da Piano Incoronata, 85048 Rotonda, Italy; 4 Department of Agricultural Sciences, University of Naples Federico II, Via Università 100, 80055 Portici, Italy

**Keywords:** *Prunus avium*, genotyping-by-sequencing, SNP markers, genetic relationships, fixation index, identity-by-state matrix, landraces

## Abstract

The sweet cherry is an important fruit species that is widespread globally. In addition to the well-known traditional and modern varieties, a myriad of landraces is present in Europe, as well as in southern Italy. This study aims to evaluate the population structure, genetic relationships, and cases of duplicate samples in a collection of 143 accessions using GBS-derived SNP markers. The genetic material under investigation includes modern commercial varieties, ancient European and American varieties, landraces, and individuals retrieved from small orchards. Some of the known varieties were genetically analyzed here for the first time. In addition, several genotypes were collected from the Basilicata region (southern Italy), an area largely unexplored for sweet cherry genetic resources. The relationships among genotypes were assessed using four different methods: allele frequency and ancestry estimation, principal component analysis, Neighbor-Joining tree, and identity-by-state estimation. The analyses returned quite congruent results and highlighted the presence of four main genetic groups, namely: (i) American varieties, (ii) the ‘Germersdorfer-Ferrovia’ cluster, (iii) the ‘Burlat’ group, and (iv) the group of Italian landraces. The main drivers of clustering were ancestry, geographical distribution, and some important traits such as self-compatibility. The sweet cherries from Basilicata, herewith examined for the first time, were mostly distributed within the group of Italian landraces, being particularly linked to the autochthonous varieties of the Campania region. However, some genotypes were outside this group, thus suggesting the introduction of genetic material from other Italian regions or from European countries. The considerable amount of American and European modern varieties analyzed are genetically very closely related, suggesting a reduced genetic basis. In addition, we highlighted the discriminating ability of SNP markers to distinguish between an original variety and its mutant. Overall, our results may be useful in defining conservation strategies for sweet cherry germplasm and developing future breeding programs to enlarge the genetic basis of commercial varieties.

## 1. Introduction

The sweet cherry (*Prunus avium* L.) is a diploid species (2n = 2x = 16) belonging to the Rosaceae family, whose fruits are highly appreciated for their organoleptic quality. In addition, cherries are rich in health-promoting components such as anthocyanins, carotenoids, quercetin, potassium, hydroxycinnamates, fiber, melatonin, and vitamin C. Thanks to these compounds, the intake of sweet cherries can prevent diabetes, cancer, Alzheimer’s disease, cardiovascular, and inflammatory diseases [1].

Europe contributes 31% to the world’s sweet cherry production. Italy is the leader in Europe and ranks sixth among the world producers after Turkey, the USA, Chile, Uzbekistan, and Iran, with a production of 107,000 tons (2015–2020 average) [2]. Several lines of evidence indicate that the cherry originated in the area around the Caspian and Black Seas [3], and cultivation expanded through Europe during the Roman Empire [4]. Although cultivated for over 2000 years, the cherry tree remained, at least until a century and a half ago, a crop relegated only to family consumption due to the perishable nature of the drupes and the transport inefficiency of the time [3]. Over the centuries, a multitude of local varieties, selected for fruit traits and well adapted to pedoclimatic conditions, have been bred in various European countries, including Italy. Most of that genetic material has been marginally cultivated, for family or local consumption, although in some Italian regions, there has been a sweet cherry industry based on landraces since the first half of the 20th century [5,6,7]. Indeed, Italy is rich in traditional sweet cherry germplasm but at high risk of genetic erosion, especially because some genotypes have a very limited diffusion or are present only in family gardens [7]. Cherry cultivation is widespread in all regions, but only Puglia, Campania, Veneto, and Emilia Romagna, which cover over 81% of the national production, represent an economically relevant fruit crop [8]. Italian sweet cherry varieties are divided into two main groups: soft-fleshed (tenerine) and firm-fleshed (duroni) cherries [9], mostly with red skin.

Information on genetic diversity is essential for developing more productive, nutritious, and resilient crop varieties as part of a breeding program with the aim of safeguarding food security to better cope with a changing environment. The use of molecular markers allows for the estimation of genetic variation among genotypes. In the last decades, several studies have been carried out to describe the diversity of sweet cherry using molecular techniques. Many of these investigations were based on simple sequence repeat (SSR) markers, for instance, to assess genetic variation in the local sweet cherry germplasm of different countries [10,11,12,13,14,15,16], including Italy [7,17,18,19].

Fernandez i Marti et al. [20] analyzed 40 single nucleotide polymorphisms (SNPs) derived from 3′ untranslated regions along with seven SSR markers to establish identity, verify parentage, and determine the relatedness of cultivars from various geographical regions. In a recent study, genetic diversity at 14 SSR loci was evaluated across a large panel of European accessions, highlighting that landraces, early selections, and modern cultivars mostly co-occurred in the groups of the population structure analysis, except for one cluster, which was mainly composed of modern cultivars from northeastern Europe [21].

Over the past decade, the development of next-generation technologies has allowed the efficient and cost-effective sequencing of plant genomes and the scoring of genome-wide SNPs. In sweet cherry, the first reference genome sequence was released for the Japanese variety ‘Satonishiki’ [22]. Subsequently, genome assembly and annotation of the cultivar ‘Big Star*’ was performed, together with the assessment of genetic diversity across 97 accessions representing three stages of the domestication and breeding processes [23].

Genome resequencing was performed to characterize genetic diversity, population structure, and allelic variation in a panel of 20 Greek genotypes and a wild accession, allowing for the identification of high-impact SNPs in genes involved in flowering time, dormancy, and response to biotic stress, thus providing useful resources for breeding programs [24].

On the other hand, a reduced representation of the genome can be achieved through Genotyping-by-Sequencing (GBS), which allows the simultaneous discovery and genotyping of thousands of SNPs to analyze germplasm diversity in a series of multiplexed samples [25,26,27,28,29].

In the present study, we compared newly collected germplasms of sweet cherry with known varieties. We used many GBS-derived SNP markers to analyze a wide collection of 143 genotypes. These included more than 100 old and modern commercial cultivars of various geographical origins, along with several Italian landraces, with the aim to assess (i) population structure, (ii) genetic relationships, (iii) the presence of duplicate samples, and (iv) the possibility to discriminate between an original variety and its mutant clones. Many of the varieties analyzed in our study had never been genotyped before, at least not using SNPs. Particular attention was devoted to local germplasm, especially from the Basilicata region (southern Italy), which has been a neglected area for the analysis of sweet cherry variation so far, to evaluate the diversity preserved even in family gardens and small orchards, and to compare this new material with known varieties.

## 2. Results

The genetic material analyzed in this study by means of SNP markers originates from a wide range of European and American countries and Asia (Iran, one variety) (Supplementary Table S1). Several known varieties have been high-throughput genotyped here for the first time.

### 2.1. Phenotypic Characterization

Supplementary Table S2 shows descriptive statistics of the analyzed traits, including means, minimum, maximum, and coefficient of variation (CV). The genotypes studied showed a considerable level of variation for all measured characters, as confirmed by the relatively high coefficient of variation, ranging from 24.98% (fruit firmness) to 55.11% (fruit shape).

### 2.2. GBS and SNP Identification

The sequencing of a starting set of 149 sweet cherry samples resulted in 486 million paired-end reads, with an average of 3.2 million reads per sample. The SNP calling returned 104,982 polymorphic sites.

The mean depth of coverage, the number of SNPs per individual, is reported in Supplementary Figures S1 and S2, and Supplementary Table S3. The distribution of the density of SNPs per chromosome in bins of 1 kb in size is shown in Supplementary Figure S3.

Transitions (Ts) were more abundant (59.21%) than transversions (Tv) (40.79%), with a Ts/Tv ratio of 1.45 (Supplementary Figure S4). SNP calling revealed that 22% of genotypes had negative values of the inbreeding coefficient F (Supplementary Figure S5). This means that many heterozygous sites were scored due to excess outbreeding or bad mapping. The various filtering processes led to a final dataset of 143 sweet cherry genotypes and 13,117 SNPs.

### 2.3. Allele Frequency and Ancestry Estimation

**Figure 1:**
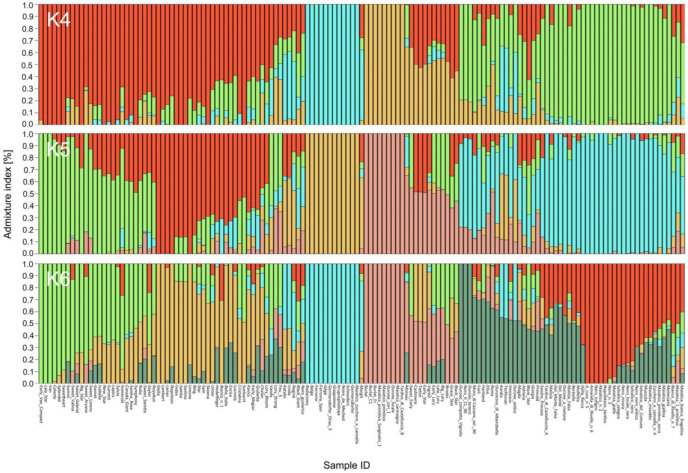
Population structure analysis based on 143 sweet cherry genotypes. Genetic clusters inferred from the population structure analysis at K = 4, K = 5, and K = 6. Names of single genotypes are indicated on the x-axis, and membership coefficient (Q) values are shown on the y-axis. Clusters at the different K values are indicated by the different colors and are named, from left to right: A, B, C, D (K = 4), A, B, C, D, E (K = 5), and A, B, C, D, E, F (K = 6). Names of genotypes are also reported in the same order as in Supplementary Table S4.

The cross-validation test (Supplementary Figure S6) indicated 13 as the best value of K, that is, the number of possible sub-populations based on the estimation of individual ancestry. However, this result returned very small sub-populations, and most of the varieties were classified as admixed. Taking into account the genetic background of the germplasm under investigation, we considered 4, 5, and 6 to be the number of ancestry components that best described the relationships among the genetic material examined here. Considering 0.7 as the threshold for the membership coefficient (Q), at K = 4, the first sub-population (4A, red in Figure 1, Supplementary Table S4) included 19 cultivars originating from Canada, while nine varieties came from the USA. Additionally, nine varieties were from Italy, but this material was obtained by crossing with Canadian varieties, from which they inherited the S4′ allele responsible for self-compatibility. Indeed, the sub-population just described included 19 out of the 25 known self-compatible varieties in the germplasm panel here analyzed (Supplementary Table S4). The 4A sub-population was divided into two sub-populations at K = 5 (5A and 5B) and K = 6 (6A and 6B). In particular, the sub-populations 5A and 6A (in green in Figure 1, Supplementary Table S4) shared almost all the varieties originating from Canada or obtained after crossing with Canadian varieties, except for ‘Isabella’ (from Italy, ‘S. H. Giant’ x ‘Stella’) and ‘Garnet’ (from USA, ‘S. H. Giant’ x ‘Bing’) with admixed ancestry (Q < 0.7) at K = 6.

The 5B (18 varieties, red in Figure 1, Supplementary Table S4) and 6B (16 varieties, yellow in Figure 1, Supplementary Table S4) sub-populations include some American and European varieties sharing common ancestors (see Supplementary Table S1). The 4B sub-population (blue in Figure 1, Supplementary Table S4) was very well conserved and corresponded exactly to sub-populations 5C (yellow) and 6C (blue). It consisted of 12 varieties belonging to the ‘Germersdorfer’-’Ferrovia’ group, with a Q > 0.99, all characterized by a very similar morphology but of different geographical origins (including one from the Basilicata region). All these varieties shared the same sterility alleles (S3, S12) when known, except for ‘Krupnoplodnaja’ (S5, S9).

The 4C sub-population (yellow in Figure 1, Supplementary Table S4), with an ancestry coefficient > 0.99, contained six local genotypes from Basilicata and three ‘Burlat’ clones, one cultivated in Italy (‘Burlat’), another obtained from a French collection (‘Burlat_Fr.’), and the third obtained in Italy by irradiation of the original French variety (‘Burlat C1′). This sub-population was well preserved at K = 5 (5D, pink) and K = 6 (6D, pink). The 4D sub-population (green in Figure 1, Supplementary Table S4) included local Italian material from the regions Basilicata, Campania, Emilia Romagna, Veneto, and Puglia. This group was maintained with a few exceptions at K = 5 (5E, green). At K = 6, these varieties were divided into two sub-populations: 6E (grey), including six varieties, and 6F (red), containing some of the varieties from Basilicata, one from Campania, and one from Puglia, while the other local varieties were admixed.

### 2.4. Principal Component Analysis

**Figure 2:**
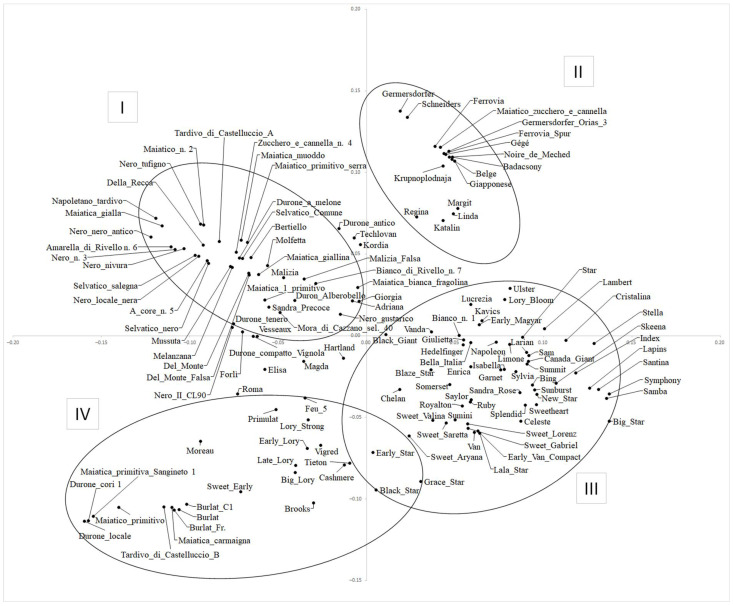
Principal Component Analysis (PCA). Diagram of the first two axes from a PCA of 143 sweet cherry genotypes. Roman numbers indicate the four main groups inferred after the analysis. I to IV indicate groups of genotypes.

The PCA plot (Figure 2) highlights the great genetic variation of the sweet cherry germplasm under investigation. Starting from the upper left portion of the plot and proceeding clockwise, four main groups can be identified. Group I includes almost all the Italian material from Basilicata, Puglia, and other regions. A smaller and fairly compact group (II) comprises ‘Germersdorfer’, ‘Shneiders’, ‘Ferrovia’, etc., corresponding to the 4B sub-population (Figure 1, Supplementary Table S4). This cluster also includes the varieties ‘Linda’, ‘Katalin’, ‘Regina’, and ‘Margit’, as they have ‘Germersdorfer’ or ‘Schneiders’ (genetically very closely related) as a parent (see Supplementary Table S1), and ‘Krupnoplodnaja’.

Most of the American varieties (from the USA and Canada) form group III, together with some modern Italian varieties, which have Canadian germplasm in their genealogy (e.g., ‘Big Star’, ‘Black Star’, ‘Blaze Star’, ‘Early Star’, etc.; Supplementary Table S1). This group contains almost all the self-fertile varieties analyzed here, e.g., ‘Celeste’, ‘Lapins’, ‘New Star’, and ‘Sweetheart’. As expected, ‘Stella’, the first self-compatible variety to be widely distributed, is included in this group. ‘Napoleon’, one of the oldest and widespread cherry varieties, is in this cluster as it has been extensively used for breeding purposes in USA and Canada. Indeed, ‘Stella’ and ‘Lambert’ (another variety widely used by American breeders) both derive from ‘Napoleon’ (Supplementary Table S1). The old varieties ‘Hedelfinger’ (Germany), ‘Bella Italia’ (Italy), and ‘Limone’ (Italy) are also present in this group since they have been found to be genetically close to ‘Napoleon’.

Group IV includes the three ‘Burlat’ sweet cherries, along with some local material from the Basilicata region, and other French (‘Early Lory’) and North American varieties (e.g., ‘Brooks’, ‘Cashmere’, and ‘Tieton’) genetically related to ‘Burlat’ (Supplementary Table S1).

Pairwise F_ST_ values were calculated between the four groups observed in the PCA plot. The highest genetic differentiation was observed between group II and group IV (F_ST_ = 0.21). The genetic distance between group I vs. group III and group I vs. group IV was the lowest (F_ST_ = 0.11). The genetic variation in all other pairwise comparisons ranged from 0.13–0.17 (Supplementary Figure S7).

### 2.5. Genetic Relationships

**Figure 3:**
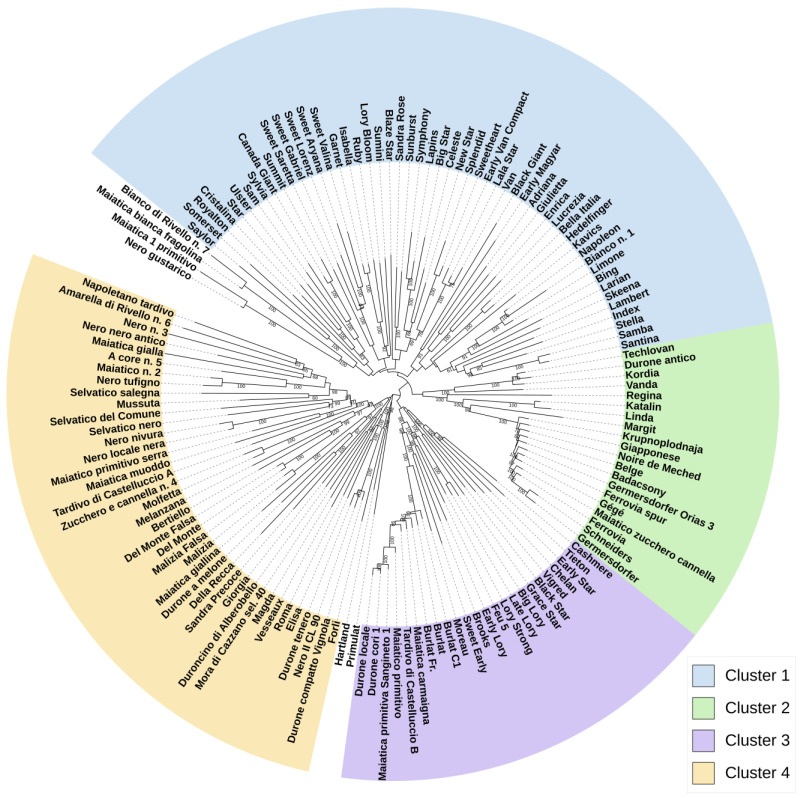
Neighbor-Joining tree obtained from SNP data on the 143 cherry genotypes. The evolutionary distances were computed using the Tajima-Nei method. Numbers on the branch nodes indicate bootstrap values (only bootstraps > 60 are shown).

The NJ tree (Figure 3) obtained using the SNP markers produced in this study provides useful information on the relationships among sweet cherry varieties and generally agrees with what is observed in the population structure and PCA analyses.

The tree is divided into four main branches. From the upper left side, apart from an external group of four genotypes from the Basilicata region (admixed in the structure analysis), the first large clade (from ‘Saylor’ to ‘Santina’, cluster 1) includes mainly American (Canada, USA) varieties and other varieties obtained using American genotypes as parents. Cluster 2 extends from ‘Techlovan’ to ‘Germersdorfers’ and includes only European varieties, with the exception of one from Iran (‘Noire de Meched’). Cluster 3, from ‘Cashmere’ to ‘Durone locale’, comprises modern varieties (from the USA and Europe), the old ‘Moreau’ and ‘Burlat’, as well as some varieties from Basilicata (Italy). Excluding the external varieties ‘Primulat’ (France) and ‘Hartland’ (USA), the fourth group (cluster 4) is the largest and contains mainly local Italian germplasm.

The main branches of the tree can be divided into subclades, in which interesting groupings can be observed. Within the North American clade, the subclade from ‘Sam’ to ‘Summit’ includes only Canadian varieties. ‘Sylvia’ and ‘Summit’ derive from ‘Van’ x ‘Sam’. ‘Canada Giant’, although of unknown pedigree, is phenotypically similar to ‘Summit’. The following subclade (from ‘Sweet Saretta’ to ‘Sweet Valina’) contains five modern Italian varieties of unknown parents, which were developed in Italy (Bologna). All these varieties are self-compatible, except ‘Sweet Valina’.

‘Garnet’ (from the USA) and ‘Isabella’ (developed in Italy) share the female parent (‘S. H. Giant’, Supplementary Table S1).

The clade from ‘Blaze Star’ to ‘Van’ is composed of Canadian and three Italian varieties (‘Blaze Star’, ‘Big Star’, and ‘Lala Star’), which have Canadian germplasm as a donor. All the varieties in this clade are related to the traditional variety ‘Van’.

‘Black Giant’ from the USA and the Hungarian ‘Early Magyar’, both of unknown origin, are early-ripening varieties [30,31].

The small subclade from ‘Adriana’ to ‘Lucrezia’ consists of Italian varieties from the Veneto region, which were developed by the same breeder, G. Bargioni [30]. In particular, ‘Giulietta’ derives from ‘Adriana’ (female parent), while ‘Enrica’ and ‘Lucrezia’ derive from ‘Vittoria’ (female parent), which, in turn, shares a common parent with ‘Adriana’ [30]. ‘Bella Italia’ (Trentino region, Italy) and ‘Hedelfinger’ (Germany) are phenotypically similar [30] and are also closely related genetically. The subclade from ‘Napoleon’ to ‘Larian’ includes three varieties closely related to each other. In fact, ‘Bing’ derives directly from ‘Napoleon’ (male parent), while ‘Larian’ derives from ‘Lambert’, which, in turn, is a seedling of ‘Napoleon’. ‘Bianco n. 1′, from the Basilicata region, and ‘Limone’, from Puglia, are genetically close to ‘Napoleon’. Apart from ‘Lambert’, the subclade from ‘Skeena’ to ‘Santina’ includes self-compatible varieties, of which ‘Stella’, deriving from ‘Lambert’, is considered the ancestor.

The following subclade groups three Czech varieties and one from Basilicata. In particular, ‘Techlovan’ and ‘Vanda’ share the same parents, ‘Van’ x ‘Kordia’. ‘Katalin’ and ‘Linda’ were developed in the same research institute in Hungary in 1990 and share ‘Germersdorfer’ as a common parent, while the German ‘Regina’ derives from ‘Schneiders’.

The subclade from ‘Krupnoplodnaja’ to ‘Germersdorfer’ includes varieties of different geographical origins but with similar phenotypes [30,31]. These varieties are genetically closely related, as already observed in the structure and PCA plots.

The varieties from ‘Cashmere’ to ‘Vigred’, from distinct geographical areas, share ‘Burlat’ as a parent, with the exception of ‘Chelan’ (USA), which has a French male parent, ‘Beaulieu’ (Supplementary Table S1). ‘Black Star’ and ‘Grace Star’ were both developed in Bologna (Italy) and share ‘Burlat’ as a parent. The subclade from ‘Big Lory’ to ‘Early Lory’ gathers all the varieties developed in France by Paul Argot in the early 1990s [31]. The American ‘Brooks’ and the Italian ‘Sweet Early’ share ‘Burlat’ as a common parent and are genetically close. The subclade from ‘Moreau’ to ‘Durone locale’ includes varieties attributable to the ‘Burlat’ group as well as some genotypes from the Basilicata region.

The following subclade groups three Italian varieties from the Emilia Romagna region (namely, ‘Forlì’, ‘Durone compatto Vignola’, and ‘Nero II CL 90′) with a genotype from Basilicata (‘Durone tenero’), in addition to two more distantly related varieties, i.e., ‘Hartland’ from the USA and ‘Primulat’ from France. The varieties from ‘Elisa’ to ‘Magda’ do not share a common geographical origin, and their genealogy is not well known.

The subclade from ‘Mora di Cazzano sel. 40′ to ‘Sandra Precoce’ assembles Italian varieties from the Veneto region, except for ‘Duroncino di Alberobello’ (from Puglia).

From ‘Della Recca’ to ‘Zucchero e Cannella n. 4′, all the Italian autochthonous varieties from Campania, together with three genotypes from Basilicata, and one from Puglia (‘Molfetta’) are included. Finally, the next subclade clade consists of all the remaining (N = 18) genotypes from Basilicata.

### 2.6. Identity by State

**Figure 4:**
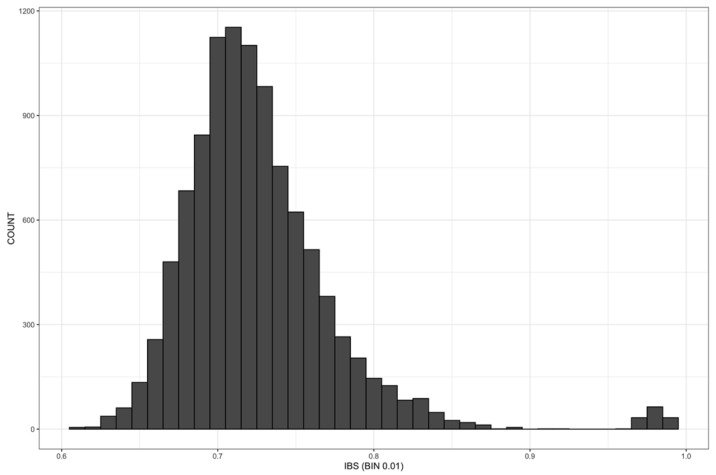
Distribution of identity-by-state (IBS) allele sharing values among 143 sweet cherry genotypes determined by the analysis of 13,117 unlinked single nucleotides polymorphisms.

Relationships among the 143 sweet cherry genotypes were also assessed in pairs by estimating identity-by-state (IBS) allele-sharing values using 13,117 unlinked SNPs. The frequency distribution of IBS estimates (Figure 4) showed that most of the genotypes fall in the bin from 0.60 to 0.97, while 75 and four pairs of individuals have allele-sharing values > 0.980 or >0.990, respectively, and no pair is completely identical (value = 1) (see Supplementary Table S5).

The highest values (>0.990) were scored for two pairs of genotypes from the Basilicata region, for ‘Del Monte’ and ‘Del Monte Falsa’, and for the pair ‘Ferrovia Spur’ and ‘Gégé’. Several pairs of genotypes with values > 0.980 are from Basilicata. Most of the pairs belonging to the ‘Germersdorfer’-’Ferrovia’ group had values between 0.980 and 0.989. Within the ‘Burlat’ group, ‘Burlat’, ‘Burlat C1′, and ‘Burlat Fr.’, as well as two genotypes from Basilicata shared values between 0.980 and 0.989. ‘Durone compatto Vignola’ and ‘Nero II CL 90′ had an IBS value of 0.985. The pairs ‘Van’-’Early Van Compact’ and ‘Van’-’Lala Star’, as well as ‘Napoleon’ and ‘Limone’, showed values > 0.980.

## 3. Discussion

In this study, we used SNP markers to assess population structure, genetic diversity, and relationships in a wide germplasm collection of 143, including old and modern commercial cultivars of different geographical origins (more than 100) and landraces mostly from southern Italy, especially newly collected material from the Basilicata region. Moreover, we investigated the possibility of ascertaining duplicate samples and discriminating between an original variety and its mutant clones. Several known varieties were genotyped here for the first time using SNPs. Others, especially the genotypes from Basilicata, are cultivated in restricted areas and lack references for both phenotypic traits and genetic characterization. The relationships among varieties/genotypes were assessed using four different methods: allele frequency and ancestry estimation, PCA, NJ tree, and IBS analysis. If we compare the results obtained by these methods, we can observe a similar trend in the grouping of varieties, determined mainly by the known genealogy of the material and/or by the geographical origin and patterns of dissemination.

Almost all the North American varieties were grouped together (Figure 1, Figure 2 and Figure 3). It has been observed that the genetic basis of modern North American varieties is very narrow because only a few founding cultivars have been used in breeding [32,33]. Among the founders, the ancient German variety ‘Napoleon’ is included in the North American cluster because it has been extensively used in the USA’s and Canadian breeding programs. ‘Napoleon’ is also a parent, directly or indirectly, of the traditional American varieties ‘Bing’ and ‘Lambert’ [34,35] and is an ancestor, from both sides, of the first self-compatible variety ‘Stella’ (group III, Figure 2; cluster 1, Figure 3). In turn, ‘Stella’ is a recurring parent in sweet cherry breeding schemes around the world, as it is the donor of the self-compatibility trait [36]. In fact, this group, essentially composed of modern genetic material from both America and Italy, includes most of the self-compatible commercial varieties, thus indicating the importance of this trait in sweet cherry breeding programs [9,35]. ‘Van’, also encompassed in this group, is a variety obtained in Canada in 1936 [9] and widely used in Canadian breeding programs. The presence of the Italian landrace ‘Limone’ (Puglia, Italy) in this cluster can be explained by the high genetic similarity and IBS value with ‘Napoleon’, as previously observed by other authors based on SSR markers [7].

The group including the varieties ‘Germersdorfer’ and ‘Ferrovia’ (group II, Figure 2; cluster 2, Figure 3) was consistently found in all the analyses carried out in this study to have high IBS values. Using AFLP markers, the following seven varieties have been proven to be genetically closely related: ‘Germersdorfer’, ‘Schneiders’ (both from Germany), ‘Badacsony’ (Hungary), ‘Noire de Meched’ (Iran), ‘Ferrovia’ (Italy), ‘Giapponese’ (Italy), and ‘Belge’ (France) [37]. That study suggested that the founder genotype of these varieties would have been renamed several times during the spread of its cultivation, probably moving from Central Europe (Germany) towards East (Hungary), West (France), and South (Italy, Iran). In particular, ‘Ferrovia’ appeared later in the Puglia region (southern Italy) in the period between 1937 and 1952. Therefore, based on various studies, ‘Ferrovia’ was considered non-autochthonous to this area, unlike what was initially believed, as it is very similar to ancient foreign varieties [37,38,39]. In the present study, we added ‘Ferrovia Spur’ (deriving from ‘Ferrovia’ by irradiation) and ‘Germersdofer Orias 3′ (a selected clone of ‘Schneiders’) [30]. Although high IBS values were observed for this group, the different genotypes appeared separated in the PCA and in the NJ tree, even for ‘Ferrovia’/’Ferrovia Spur’ and ‘Schneiders’/’Germersdofer Orias’ 3. Noteworthy is the presence of ‘Krupnoplodnaja’ in this group, sharing phenotypic traits with the ‘Germersdorfer’-’Ferrovia’ varieties (Supplementary Table S2), even though the genealogy and S-genotype reported for ‘Krupnoplodnaja’ appear to be unrelated to the remaining cultivars in this cluster [21,34].

‘Burlat’ is an ancient French variety, much appreciated and widespread globally, thanks to its early ripening and other quality traits [9]. For this reason, ‘Burlat’ has been extensively used in breeding programs, giving rise to other known varieties, e.g., ‘Sweet Early’, ‘Primulat’, and ‘Early Lory’ (Supplementary Table S1). These latter cultivars, although with admixed ancestry, can be included in the ‘Burlat’ group (see Figure 2, group IV, and Figure 3, cluster 3), which also encompasses the ancient French ‘Moreau’, a variety phenotypically similar to ‘Burlat’ and developed in the same French region, Rhône [40], thus suggesting a correlated ancestry with ‘Burlat’. The variety ‘Chelan’, derived from ‘Stella’ x ‘Beaulieu’, is included in the group of American varieties in the PCA plot (Figure 2), while it is included in the ‘Burlat’ group in the NJ tree (Figure 3). This could be explained considering that the French ‘Beaulieu’, although of unknown origins, could be related to ‘Burlat’, given the widespread dissemination of this latter variety in France [40]. This ‘Burlat’ group also includes some genotypes from Basilicata, probably due to the exchange of material among regions and introduction from abroad, as already observed in other studies for other landraces [7], considering that ‘Burlat’ was introduced in Italy in the first half of the 20th century [41]. All the analyses in this study allowed us to distinguish ‘Burlat’, ‘Burlat C1′, and ‘Burlat Fr’.

Based on the estimate of the F_ST_, the highest genetic differentiation was observed between the ‘Burlat’ group (from France; group IV in Figure 2; cluster 3 in Figure 3) and the ‘Germersdofer’ group (from Germany; group II in Figure 2; cluster 2 in Figure 3), probably due to the low number of genotypes in each of these groups and the close genetic relatedness of the varieties in each group (see Table S1). Indeed, it was found that the ancient European varieties could be clearly separated based on their geographical origin [42].

Almost all the Italian landraces and old varieties are grouped together and, in general, are placed in the NJ tree according to their geographical distribution (cluster 4, Figure 3), as suggested in previous studies [7].

‘Malizia’ and ‘Malizia Falsa’ (group I in Figure 2; cluster 4 in Figure 3) were considered synonyms based on SSR markers [7]; however, our results showed an IBS value lower than 0.980, and therefore, these two varieties can be distinguished using SNP markers. Indeed, phenotypically, these two varieties differ in early (‘Malizia Falsa’) or intermediate (‘Malizia’) ripening time [43]. Therefore, compared to SSRs, SNP markers confirmed a greater power for discriminating mutants from their original parents [20].

The sweet cherry varieties from the Campania region cluster with most of the germplasm from Basilicata. It has been hypothesized that cherry cultivation was started in Greece in 300 B.C. [44] and that this crop was introduced in Italy by the ancient Romans in 73 B.C. [45]. However, it seems that cherry was cultivated in Italy long before that date [3,45], and hundreds of varieties have developed over the centuries. In a recent survey, over 700 names of local sweet cherry varieties have been recorded in Italy, with a prevalence in southern regions, except for Basilicata, which was not well represented in that study [43]. Indeed, ancient Romans appreciated sweet cherries, which appear in Roman frescos of Pompeii, Campania (II century BC) [46]. Therefore, given the long-term spread of cherry cultivation in Campania and the geographical proximity to Basilicata, it is highly probable that there has been a transfer of genetic material from the former to the latter region, whose cherry genetic resources were almost completely unexplored.

## 4. Materials and Methods

### 4.1. Plant Material, Phenotypic Characterization, and Genotyping-by-Sequencing

The sweet cherry germplasm used in this study (Supplementary Table S1) was obtained from the field collection managed by the University of Bari and located in Valenzano (Italy), from the field collection run by the “Agenzia Lucana di Sviluppo e di Innovazione in Agricoltura” (ALSIA) and located in Rotonda (Italy), and from various orchards located in the Basilicata region (Italy).

The selected varieties were subjected to phenotypic characterization, and five essential traits were observed: fruit shape, skin color, flesh color, flash firmness, and ripening time. Phenotyping was carried out for two consecutive years (2018–2019) according to sampling and classification protocols reported by UPOV guidelines [47].

Leaves were collected at the end of the summer season, and healthy tissues were used for genomic DNA isolation and purification using the DNeasy plant mini kit (Qiagen, Milano, Italy).

The quality and concentration of each DNA sample were assessed before shipping the samples to the Elshire Group Ltd. (Palmerston North, New Zealand) for GBS assay. Since the ApeKI restriction enzyme had already been used successfully to generate GBS libraries in *Prunus* [48], this enzyme was chosen to cut up DNA into fragments. Enzymatic digestion of DNA, DNA amplification, and library preparation were performed according to [25]. Briefly, for each sample, 100 ng of genomic DNA and 3.6 ng of total adapters were used. The PCR-amplified sequence library (18 cycles) was sequenced in paired-end mode (2 × 100 bp) on an Illumina HiSeq (San Diego, CA, USA) device.

### 4.2. Preprocessing of GBS Tags, SNP Calling, and Filtering

Raw reads were demultiplexed with axe-demux (https://github.com/kdmurray91/axe; accessed on 20 December 2021), and technical Illumina sequences were removed. High-quality reads were then aligned to the *P*. *avium* reference genome (https://www.rosaceae.org/species/prunus_avium/genome_v1.0.a1; accessed on 20 December 2021) with Bowtie2 [49] in end-to-end mode, sensitive. R1 and R2 reads were aligned independently, merged in bam files, and sorted by mapping position. The reference-based SNP calling pipeline in STACKS 2.3 (http://catchenlab.life.illinois.edu/stacks/; accessed on 20 December 2021 [50]) was used to identify SNP loci. The raw VCF (variant call format) file was processed using VCFtools (version 0.1.17; [51]) with the following filtering options: minimum allele frequency (MAF) ≥ 0.05, max-missing = 0.80, and min-mean depth = 5. InDels were removed. Several functions in VCFtools were used to generate statistics on the dataset under investigation. Individuals with more than 20% missing genotype data were filtered out using PLINK v1.90b6.24 [52]. The PLINK—indep-pairwise command (window size = 50 kb; step size = 5; r2 = 0.5) was used to generate a pruned subset of SNP markers in approximate linkage equilibrium with each other.

### 4.3. Population Structure and Genetic Relationships

The relationships among genotypes were assessed using four different methods: identity-by-state estimation, allele frequency and ancestry estimation, principal component analysis, and Neighbor-Joining tree.

PLINK was used to populate the matrix of genome-wide IBS (identity-by-state) pairwise distances. Pairwise F_ST_ values between populations were computed using the fst.hudson function as part of the KRIS v1.1.6 software package (https://CRAN.R-project.org/package=KRIS; accessed on 3 January 2022 [53]). The fst.hudson function is based on Hudson’s description [54] and Hudson’s estimator [55]. A heatmap visualizing pairwise F_ST_ values was built using ggplot2 v3.3.6 (https://CRAN.R-project.org/package=ggplot2; accessed on 3 January 2022).

An ADMIXTURE version 1.3.0 [56] was performed, assuming ancestral populations (K) between 1 and 20. The parameters -cv = 10, -B1000, and -c 5. Cross-validation (CV) scores were used to determine the best K value. Individuals were assigned to a specific sub-population when the membership coefficient (Q) was ≥0.7; otherwise, they were considered admixed.

Principal component analysis (PCA) was used on the pruned SNP dataset to describe the overall population structure and derive patterns of relatedness between individuals.

The same SNP dataset was used to generate the Neighbor-Joining (NJ) tree based on the Tamura-Nei genetic distance model [57] using MEGA X (https://www.megasoftware.net/; accessed on 10 March 2022 [58]) with 500 bootstrap replicates. The tree was imported and annotated with iTOL v6 (https://itol.embl.de/; accessed on 10 March 2022 [59]).

## 5. Conclusions

SNP markers obtained via GBS proved to be an excellent tool for assessing the genetic diversity and relationships within sweet cherry, consistent with pedigree and/or geographical origin when applied to a large and well-assorted germplasm panel. The present study highlights that the significant number of American and European modern varieties analyzed here are genetically closely related, suggesting a reduced genetic basis. Moreover, SNPs were able to discriminate between varieties previously considered synonyms (‘Malizia’ and ‘Malizia Falsa’) and between varieties and their selected clones (e.g., ‘Burlat’ and ‘Burlat C1′; ‘Ferrovia’ and ‘Ferrovia Spur’). Additionally, we were able to confirm some relationships previously observed using different types of molecular markers, e.g., the genetic similarity between ‘Napoleon’ and ‘Limone’, as well as the high similarity among the varieties included in the ‘Germersdorfer’-’Ferrovia’ group, although these varieties could be distinguished, confirming the discriminant power of the SNP markers.

Furthermore, for the first time, we have explored the germplasm from Basilicata (southern Italy), highlighting that most of this material has genetic affinities with landraces from other Italian regions, especially Campania, from which genetic material could have been transferred. In addition, some of the genotypes from Basilicata appear more closely related to foreign varieties, suggesting that this genetic material could derive from the local adaptation of ancient European varieties, which probably arrived in southern Italy several decades ago.

The conservation and availability of variable germplasm preserved over time can warrant the retention of a potentially useful gene sink for future breeding programs, broadening the genetic basis of commercial cultivars. Furthermore, local varieties should also be preserved to ensure production and marketing in the communities where they were developed.

Finally, the availability of SNP markers for a wide panel of individuals could guarantee the reuse of data for identifying SNP loci associated with key agronomic traits.

## Data Availability

The raw sequence data from this study were deposited in the NCBI database with the bioproject accession number: PRJNA865379. The SNP data are available on “figshare” at the link: https://dx.doi.org/10.6084/m9.figshare.21571803 (accessed on 20 October 2022).

## Supplementary Materials

The following supporting information can be downloaded at: https://www.mdpi.com/article/10.3390/plants12010136/s1, Figure S1: Bar chart showing the mean depth per-individual basis; Figure S2: Bar chart showing the SNP count per-individual basis; Figure S3: Distribution of the density of SNPs per chromosome in bins of 1 kb in size; Figure S4: Bar chart describing the distribution of SNP types; Figure S5: Bar chart of the inbreeding coefficient F computed per-individual basis; Figure S6: Cross-validation scores used to determine the best K value; Figure S7: Heatmap of the pairwise F_ST_ values between sub-populations. Numbers 1, 2, 3, and 4 indicate the groups of Italian landraces, ‘Germersdorfer’-‘Ferrovia’, American varieties, and ‘Burlat’, respectively; Table S1: Sweet cherry genotypes, country of origin, presumed parentage, and S alleles; Table S2: Phenotypic traits of sweet cherry cultivars according to UPOV (1995) descriptors and descriptive statistics; Table S3: Mean depth and SNP count on an individual basis for each chromosome. The coefficient of variation is shown for both metrics; Table S4: Individual membership coefficients (Q) values of each genotype inferred from the population structure analysis at K4, K5, and K6. Colors indicate the sub-populations (SUB-POP) to which varieties belong, considering a threshold of Q ≥ 0.7 (darker color). The non-colored lines indicate admixed samples; Table S5: Identity-by-state allele-sharing values for pairs of sweet cherry varieties. Only values > 0.980 are shown.
