# Supplementary figures and images for: Sweet Cherry Diversity and Relationships in Modern and Local Varieties Based on SNP Markers
Source: Plants (Basel). 2022 Dec 27;12(1):136. doi: 10.3390/plants12010136 (PMC9824393; doi:10.3390/plants12010136)

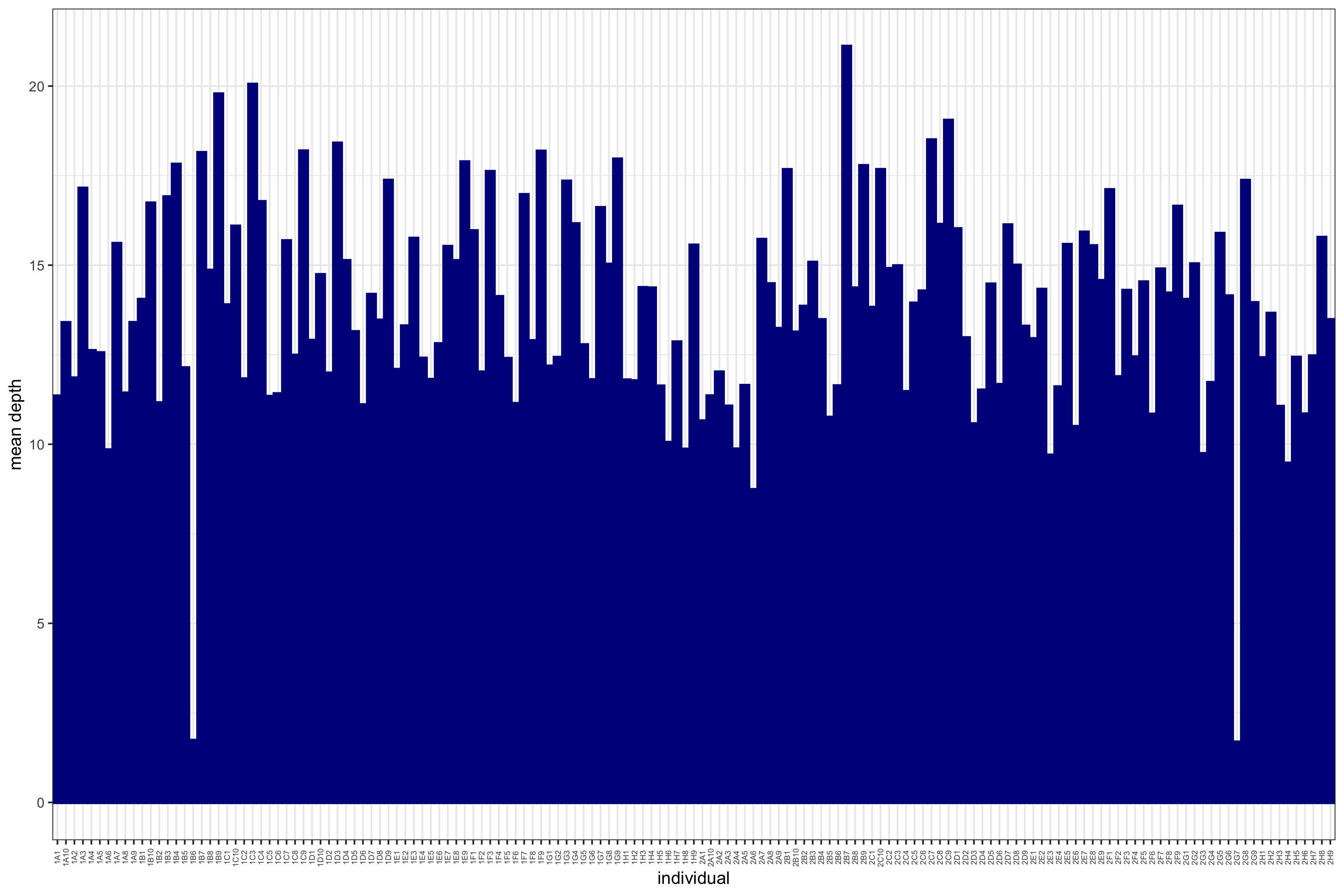

Supplement: Supplementary file 1 [file plants-12-00136-s001.zip › Suppl_FigureS1.jpg]

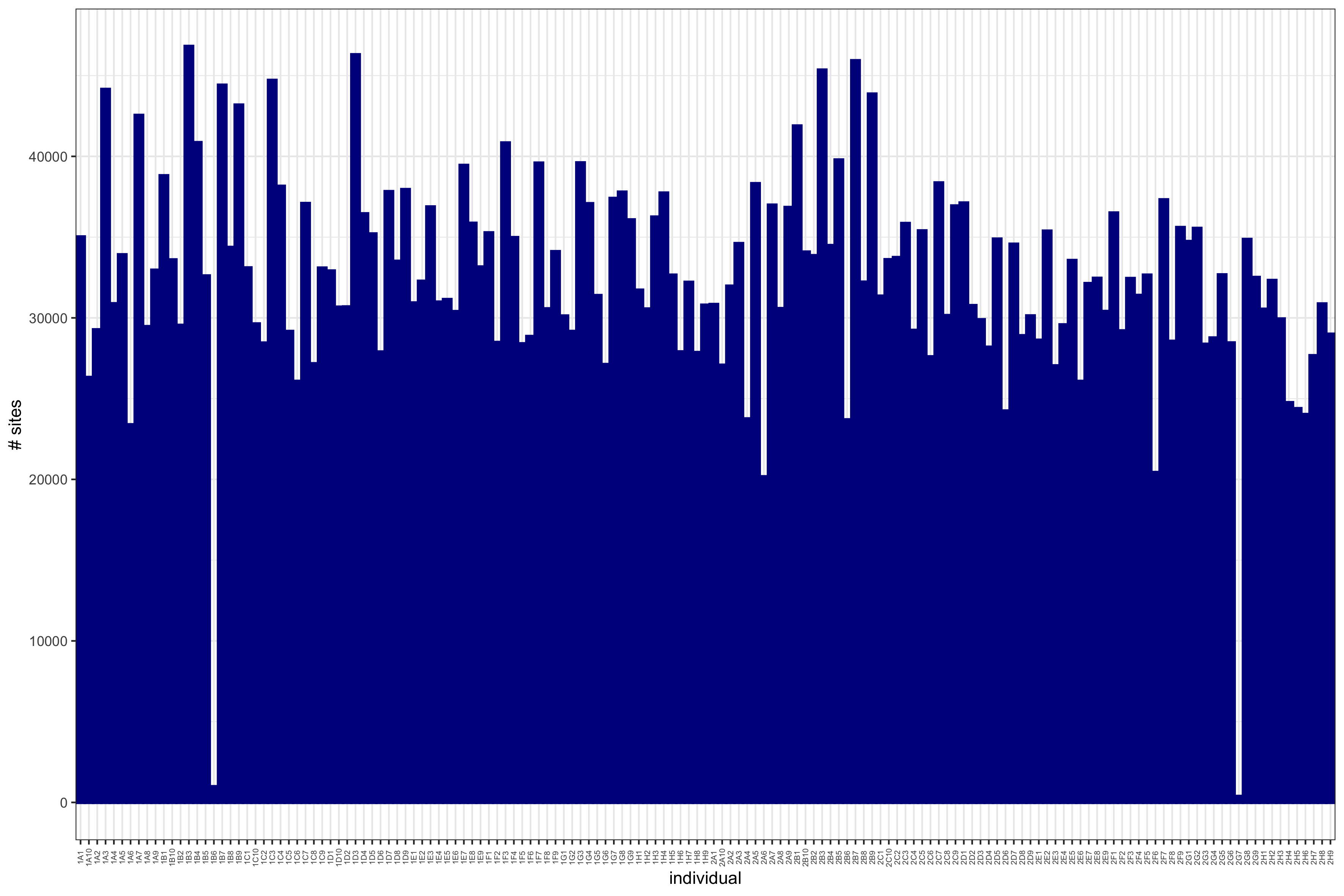

Supplement: Supplementary file 1 [file plants-12-00136-s001.zip › Suppl_FigureS2.jpg]

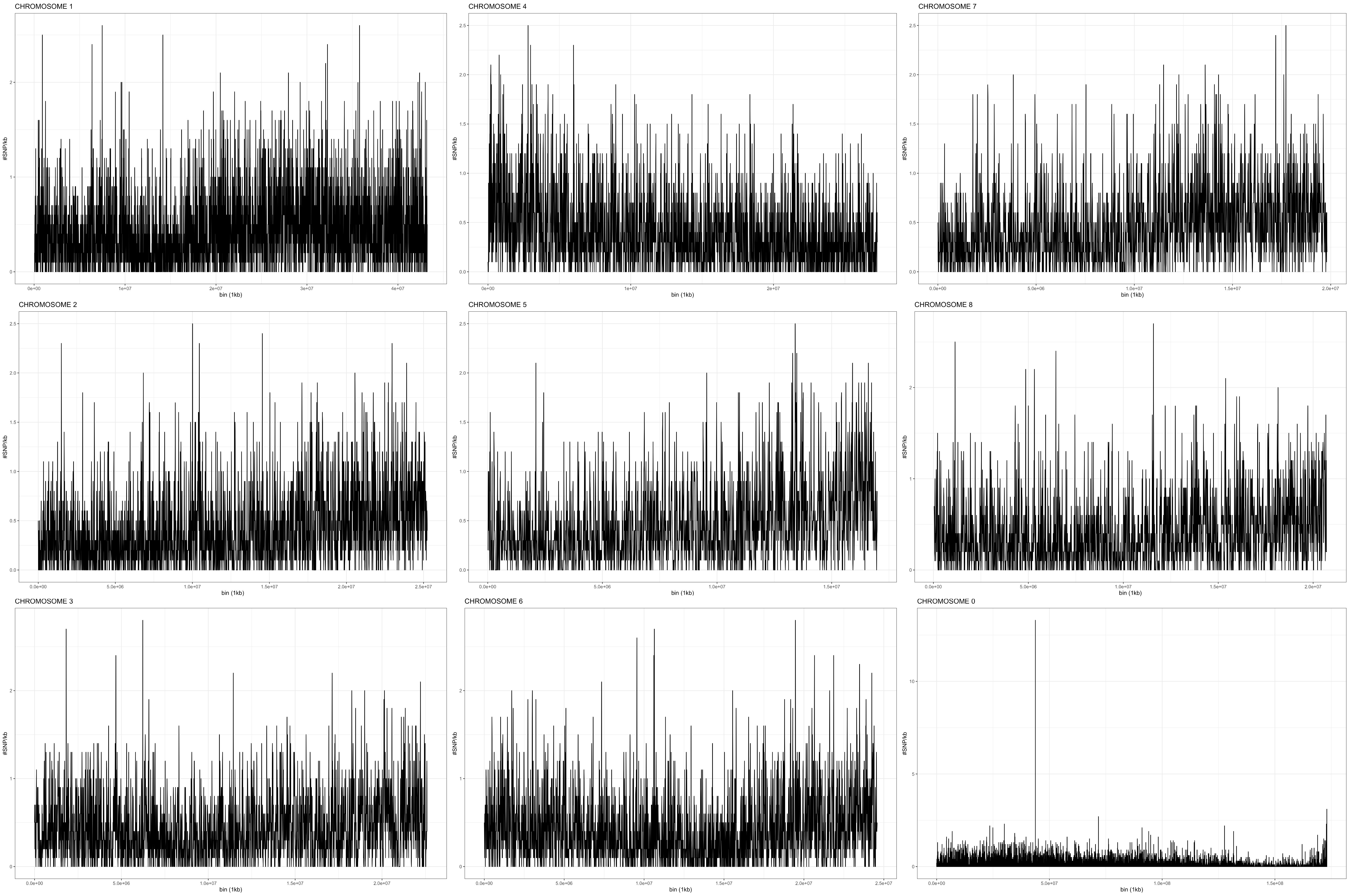

Supplement: Supplementary file 1 [file plants-12-00136-s001.zip › Suppl_FigureS3.jpg]

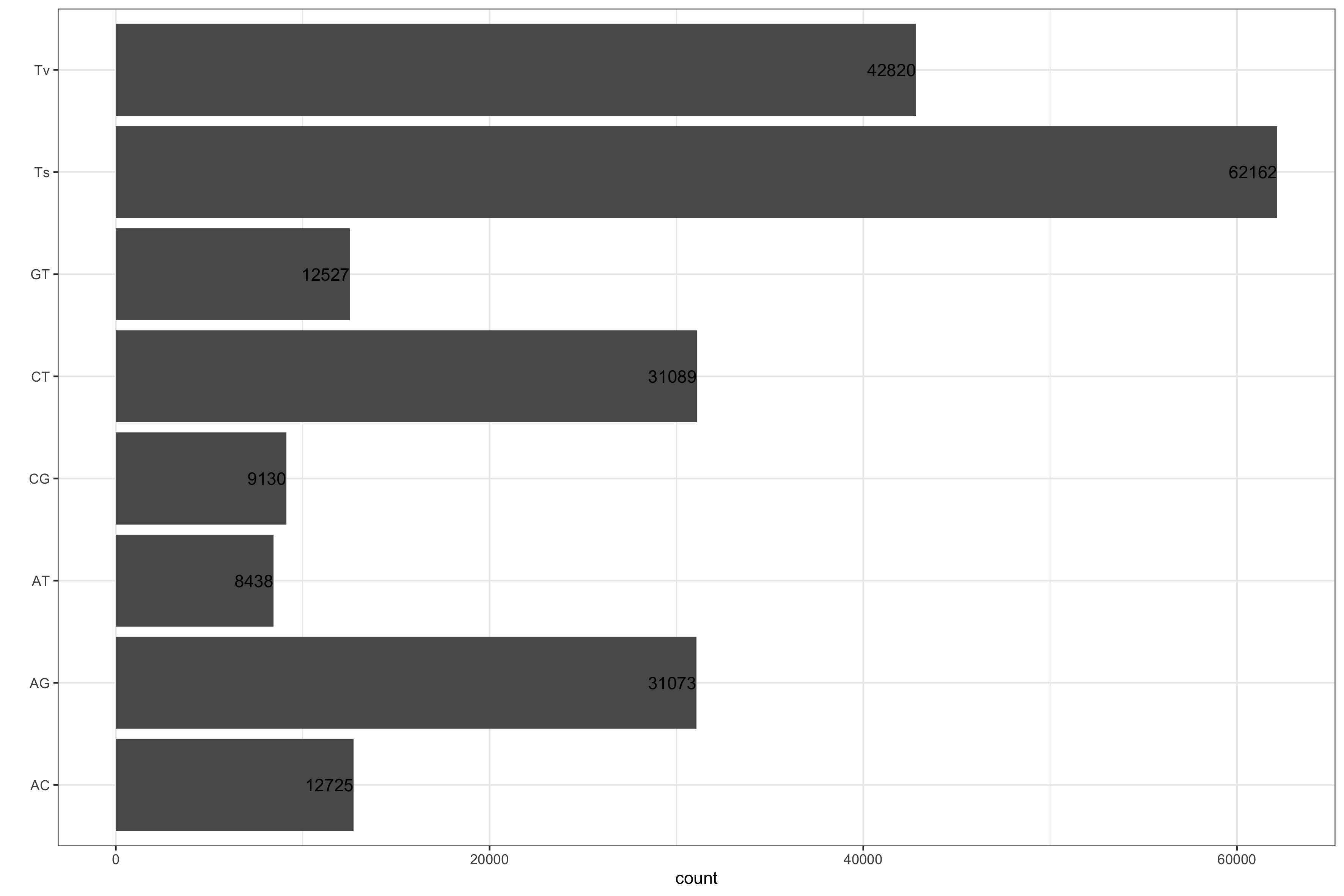

Supplement: Supplementary file 1 [file plants-12-00136-s001.zip › Suppl_FigureS4.jpg]

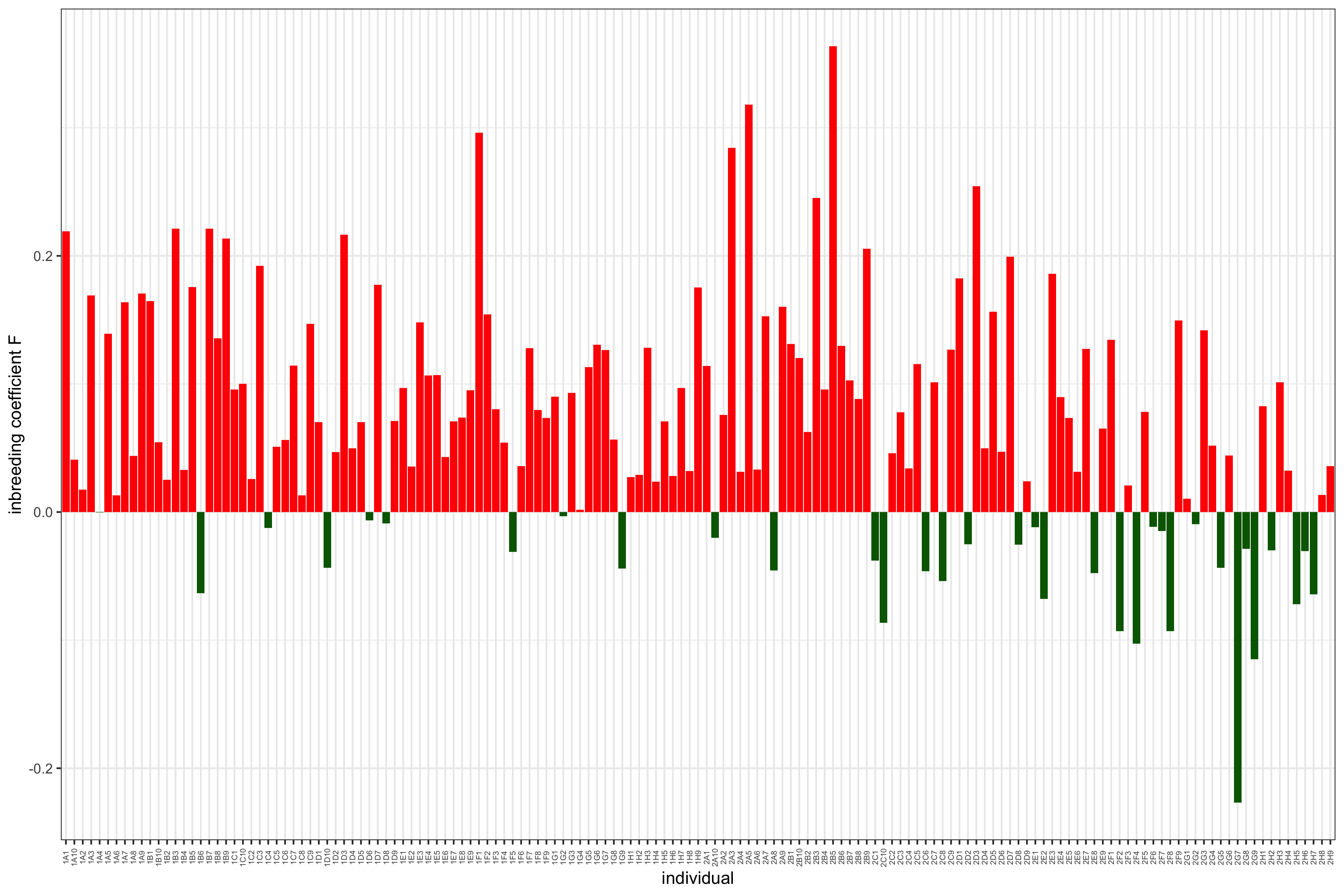

Supplement: Supplementary file 1 [file plants-12-00136-s001.zip › Suppl_FigureS5.jpg]

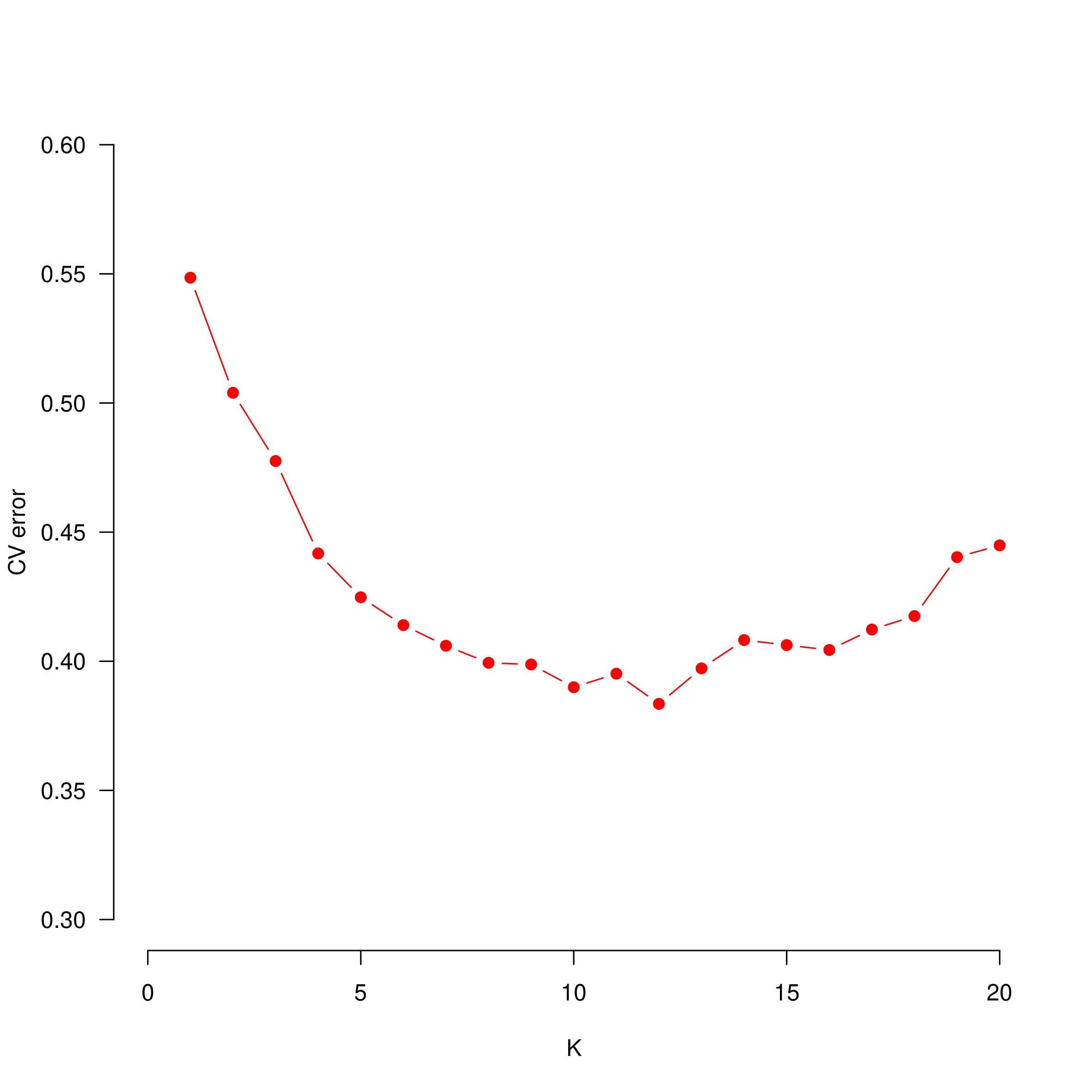

Supplement: Supplementary file 1 [file plants-12-00136-s001.zip › Suppl_FigureS6.png]

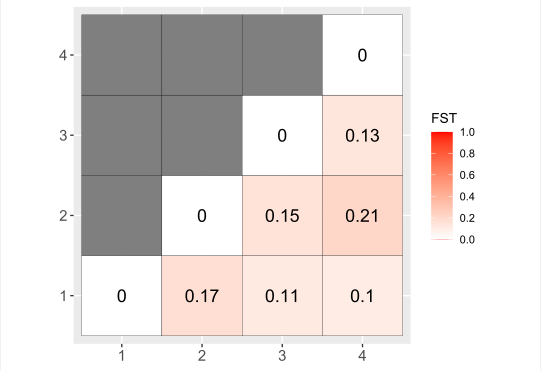

Supplement: Supplementary file 1 [file plants-12-00136-s001.zip › Suppl_FigureS7.tif]
